# Lower-limb coordination during the rear-leg roundhouse kick in competitive taekwondo: a vector-coding analysis

**DOI:** 10.1186/s13102-026-01831-5

**Published:** 2026-07-31

**Authors:** Qi Guo, Yong Ma, Lin Liu, Gan Liu, Mengyao Jia

**Affiliations:** 1 School of Intelligent Sports Engineering, Wuhan Sports University, No. 461 Luoyu Road, Wuhan, Hubei Province 430079 China; 2 Key Laboratory of Sports Engineering of General Administration of Sports of China, Wuhan Sports University, Wuhan, 430079 China; 3 Engineering Research Center of Sports Health Intelligent Equipment of Hubei Province, Wuhan Sports University, Wuhan, 430079 China; 4 Specialised Research Centre for High-Quality Development of Competitive Sports, Wuhan Sports University, Wuhan, 430079 China; 5 School of Education (Normal School), Dongguan University of Technology, Dongguan, 523808 China; 6 Sports and Health Industry Science and Technology Innovation Center, Dongguan University of Technology, Dongguan, 523808 China

**Keywords:** Motor coordination, Dominant side, Non-dominant side, Rear-leg roundhouse kick, Vector coding

## Abstract

**Objective:**

To investigate whether differences exist in coupling angle (CA)-derived joint coordination pattern characteristics and coordination variability between the dominant side (DS) and non-dominant side (NS) during successful scoring rear-leg roundhouse kick (RLRK) in competitive taekwondo athletes.

**Methods:**

Twelve male taekwondo athletes were recruited to perform RLRK with both the DS and NS. Lower-limb kinematic data were collected using a Vicon three-dimensional motion capture system. Signals from the Daedo electronic protector and a Kistler force platform were used for valid trial selection and movement event identification. Custom-written MATLAB code was used to calculate CA trajectories and coordination variability for lower-limb joint couplings. CA data were analyzed using circular statistics, with mean CA used to characterize CA-derived joint coordination pattern characteristics, and coordination variability was compared using paired-samples *t*-tests.

**Results:**

No significant bilateral differences in mean CA were observed during the knee-lift phase. The bilateral differences in mean CA were mainly observed during the kicking and recovery phases, reflecting side-related differences in coordination characteristics represented by CA. Specifically, significant bilateral differences were found in the frontal-plane hip–ankle coupling and sagittal-plane knee–ankle coupling during the kicking phase *(p* = *0.037; p* = *0.026)*, and in the sagittal-plane knee–ankle coupling during the recovery phase *(p* = *0.016)*. Significant side-related differences in coordination variability were observed across all three movement phases. During the knee-lift and kicking phases, significant differences were found in the sagittal-plane knee–ankle coupling *(p* = *0.002; p* = *0.015*). During the recovery phase, significant bilateral differences were observed in the frontal-plane hip–ankle coupling *(p* = *0.008*), sagittal-plane hip–knee coupling *(p* = *0.027*), and sagittal-plane knee–ankle coupling (*p* = *0.044*).

**Conclusions:**

Competitive taekwondo athletes exhibit side-specific lower-limb joint coordination control strategies when executing RLRK. These differences were reflected in mean CA-based coordination pattern characteristics and coordination variability. Lower coordination variability in certain joint couplings on the DS during the knee-lift and kicking phases suggests that the DS may adopt a relatively more stable coordination control strategy during the key output stages of the movement. In the recovery phase, bilateral differences were mainly reflected in joint-specific coordination organization and in the distribution of coordination variability.

**Supplementary Information:**

The online version contains supplementary material available at https://doi.org/10.1186/s13102-026-01831-5.

## Introduction

In competitive taekwondo, kicking techniques constitute a key factor in determining competitive performance because of their pronounced offensive effectiveness, high target-hit rate, and substantial scoring potential [1]. The rear-leg roundhouse kick (RLRK), in particular, is frequently employed in both offensive and counteroffensive contexts because of its high striking power, extensive attack range, and deceptive execution [2]. The widespread use of electronic protectors has further clarified and standardized the scoring criteria for this technique. Specifically, athletes are required to strike a valid scoring area accurately and generate sufficient impact force to surpass the predefined scoring threshold [3]. Successful execution of the RLRK relies on finely coordinated interactions among multiple lower-limb joints, and effective joint coordination is essential for optimizing force production [4], speed control [5], and directional precision [6], all of which directly influence athletic performance. From a biomechanical perspective, the RLRK is particularly suitable for multi-joint coordination analysis because it is a rapid, sequential, and phase-dependent kicking movement. Its execution requires the continuous participation of the hip, knee, and ankle joints, with force and motion being transmitted along the lower-limb kinetic chain toward the distal striking segment. Therefore, an analytical framework based solely on single-joint characteristics may be insufficient for capturing the dynamic organization of lower-limb movement during this technique.

Vector coding is an analytical approach used to quantify inter-joint coupling by calculating the orientation changes of vectors formed between consecutive points on an angle-angle plot, thereby enabling continuous tracking of dynamic interactions across different phases of movement [7, 8]. More importantly, because the RLRK involves continuous changes in proximal–distal joint relationships rather than isolated joint motions, the central methodological challenge is to identify how lower-limb joints are coordinated across successive time points and movement phases. Compared with conventional single-joint kinematic analyses, vector coding is more suitable for capturing the dynamic characteristics of multi-joint coordinative control during complex motor tasks [9, 10]. Previous studies have applied this method to examine coordination patterns and coordination variability in gait, running, and functional ankle instability, demonstrating its value as an effective tool for investigating coordinative control characteristics in human movement [11–13]. Given that the RLRK involves rapid transitions from knee lift to kicking and then to recovery, vector coding provides a suitable framework for examining how adjacent lower-limb joints interact continuously rather than only describing isolated peak angles or angular velocities. Accordingly, in the context of the present study, vector coding was not used as an additional descriptive analysis, but as the core analytical tool for addressing whether bilateral differences between the dominant side(DS) and non-dominant side (NS)are reflected in phase-specific lower-limb coordination patterns and coordination variability.

During sport-specific training, athletes typically develop a clear preference for a dominant or primary kicking leg, resulting in side-specific tendencies in kicking tasks [14]. Over time, the cumulative effects of asymmetric training loads may give rise to bilateral differences in movement proficiency, neuromuscular control, and motor performance, which may in turn increase injury risk and compromise execution accuracy [15, 16]. In taekwondo, such bilateral asymmetry may affect not only the consistency of technical performance but also the organization and stability of multi-joint coordination during the RLRK. Therefore, examining joint coordination characteristics during the RLRK from a bilateral comparative perspective is of clear practical relevance for taekwondo training and performance optimization.

At present, research on taekwondo techniques has primarily focused on discrete kinematic, kinetic, and electromyographic variables, such as reaction time, execution time, impact force, joint angular velocity, joint moment, and muscle activation. However, direct continuous time-series evidence remains limited regarding inter-joint coordination during the RLRK, particularly with respect to phase-specific bilateral differences between the DS and NS. This limitation is important because the knee-lift phase mainly involves limb preparation and positioning, the kicking phase requires rapid distal acceleration and impact generation, and the recovery phase requires post-impact limb repositioning and movement stabilization. These different functional demands make the RLRK an appropriate model for examining continuous hip–knee–ankle coordination across movement phases. Therefore, the present study used vector coding to compare lower-limb joint coordination patterns and coordination variability when competitive taekwondo athletes performed the RLRK with the DS and NS, with the aim of identifying bilateral differences in joint coordination organization across movement phases. The main contribution of this study is the application of vector coding to reveal phase-specific bilateral coordination differences during the RLRK in competitive taekwondo. We hypothesized that phase-specific differences in coordination patterns and coordination variability would exist between the DS and NS during the RLRK, and that such differences would be more pronounced during the kicking phase.

## Materials and methods

### Participants

The required sample size was estimated using G*Power 3.1.9.2. Based on a large effect size (*d* = 0.8), a significance level of α = 0.05, and a statistical power of 80%, the minimum sample size was calculated to be 12 participants. Accordingly, 12 male taekwondo athletes from Wuhan Sports University were recruited for this study. To protect participant anonymity, individual-level anthropometric and training-experience data are not reported. At the group level, the participants had a mean age of 18.25 ± 2.09 years, a mean height of 182.83 ± 8.63 cm, a mean body mass of 71.42 ± 14.53 kg, and systematic taekwondo training experience of 4.75 ± 0.97 years (Table 1). All participants had obtained at least a National Second-Class Athlete certificate. In addition, all participants had formal competition experience at the provincial or national level, indicating that the sample represented trained competitive taekwondo athletes rather than recreational practitioners. The inclusion criteria were as follows: (1) held a National Level 2 athlete certificate or above; (2) having systematic taekwondo training experience of at least 4 years and formal competition experience; (3) no lower-limb sports injury within the previous 6 months; (4) regular taekwondo training with an average frequency of approximately five sessions per week, with each session lasting about 3 h; and (5) no high-intensity training within 24 h before testing to ensure an adequate physical condition during the experiment. All participants provided written informed consent prior to participation. For participants younger than 18 years, written informed consent was obtained from their legal guardians, and written assent was also obtained from the participants themselves before participation. This study was conducted in accordance with the Declaration of Helsinki and was approved by the Ethics Committee of Wuhan Sports University (Approval No. 2020048).

**Table 1:** Subject information summary

Characteristic	Summary
Sample size	12
Sex	Male
Age (years)	18.25 ± 2.09
Height (cm)	182.83 ± 8.63
Body mass (kg)	71.42 ± 14.53
Training experience (years)	4.75 ± 0.97
Competitive level	National Second-Class Athlete certificate or above
Competition experience	Provincial or national level

### Experimental methods

#### Definition of the dominant side

Because of side preference and functional specialization developed through long-term sport-specific training, athletes can typically be classified according to a dominant side (DS) and a non-dominant side (NS), with the dominant lower limb also referred to as the dominant leg, preferred leg, or primary kicking leg [17]. In the present study, the dominant leg was defined as the lower limb primarily used by the athlete to perform the RLRK during routine training and competition. This classification was determined based on the athlete’s self-report and was further confirmed by the coach. In addition, the preferred kicking side observed during the familiarization and experimental trials was checked to ensure consistency with the self-reported dominant leg. If any inconsistency had been observed among self-report, coach confirmation, and experimental observation, the classification would have been rechecked before data analysis. Among all participants, the dominant leg was the left leg in 3 athletes and the right leg in 9 athletes.

#### Experimental equipment

**Fig. 1:**
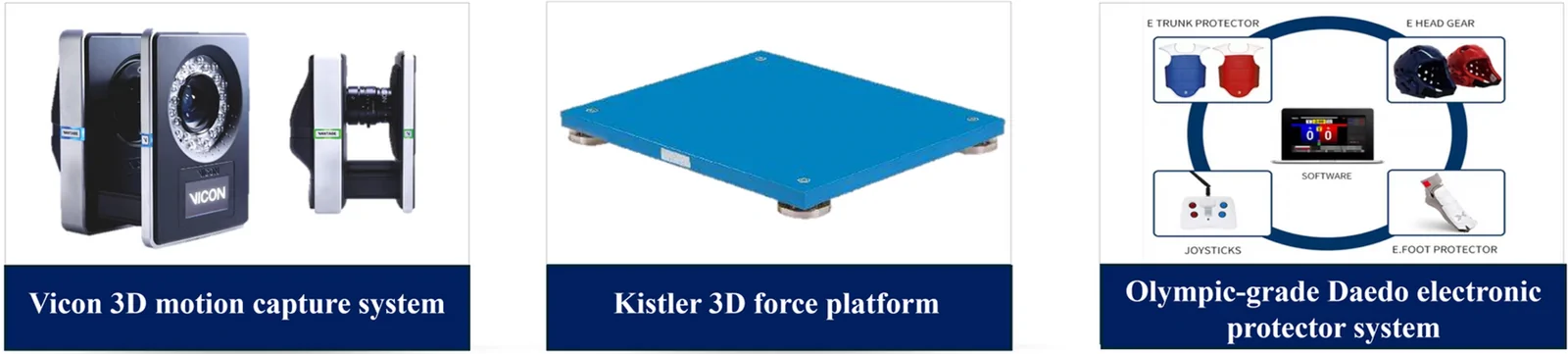
Experimental apparatus

Raw kinematic data were collected using a nine-camera infrared high-speed motion capture system (T40, Vicon, Oxford, UK) at a sampling frequency of 200 Hz. Kinetic data were recorded using four three-dimensional force platforms (Kistler, Switzerland) at 1,000 Hz. The Vicon and Kistler systems were synchronized via a digital signal converter. An Olympic-standard Daedo electronic protector system was used to determine whether each RLRK resulted in a valid score. In combination with force platform signals, this system was also used to identify key movement events and thereby define the movement phases (Fig. 1).

#### Test preparation and procedure

**Fig. 2:**
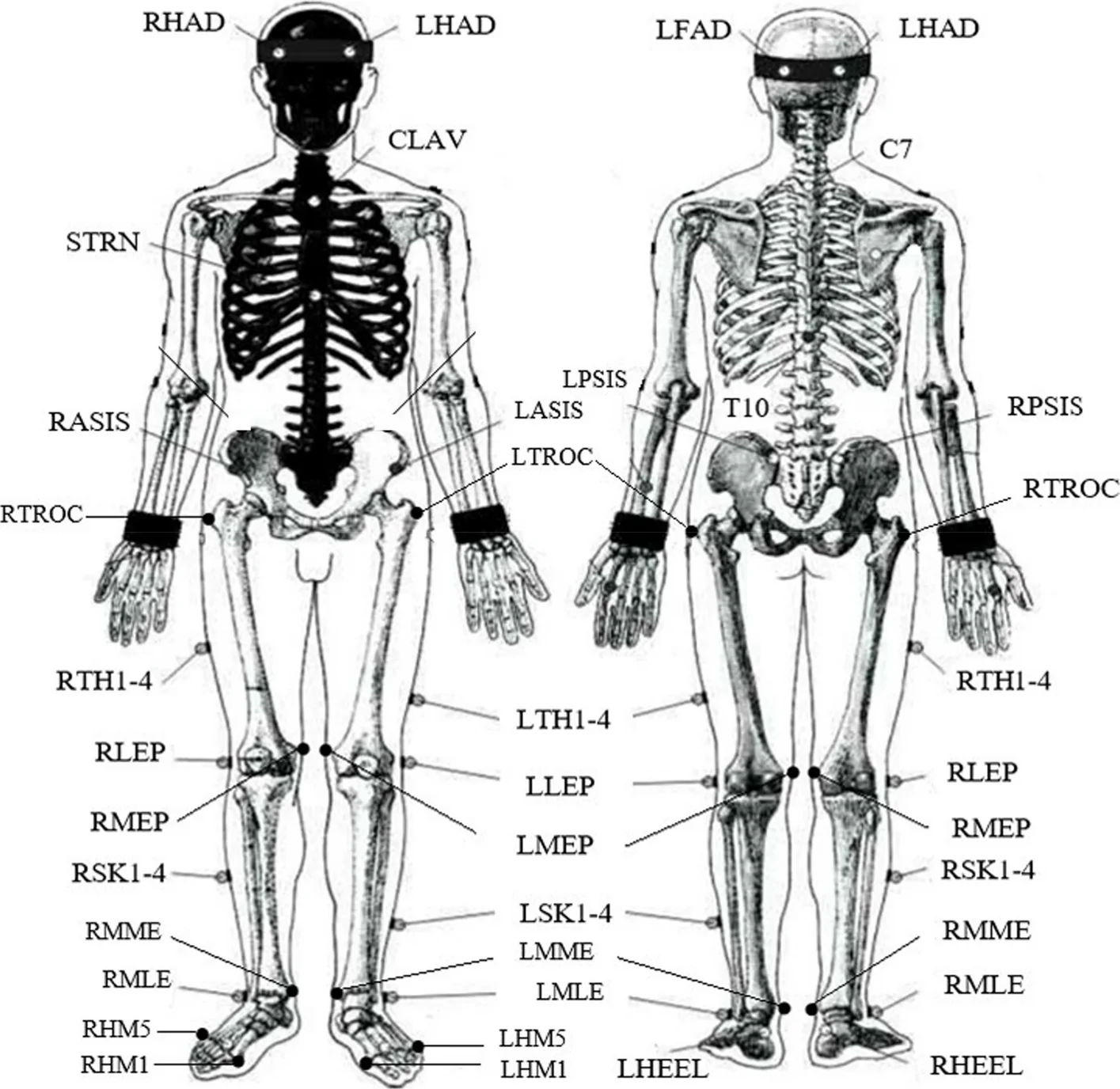
Schematic diagram of the experimental marker placement

Testing was conducted in the Key Laboratory of the General Administration of Sport of China. After changing into standardized sportswear, participants first completed 5 min of whole-body stretching, with emphasis on the lower-limb muscle groups. The stretching protocol included static stretching of the hamstrings, quadriceps, triceps surae, and hip flexors, with each stretch held for 30 s per side. Participants also performed dynamic stretching exercises, including forward and backward leg swings, lateral leg swings, rotational lunges, and hip circumduction, to increase joint mobility and prepare for the subsequent testing procedure. After stretching, participants performed 5 min of treadmill jogging at 6.5 km·h^1^ with a 0% incline, followed by an additional 5 min of stretching according to the standard pre-training warm-up routine, in order to further reduce the risk of sports injury during the experiment [3, 18]. After the warm-up, reflective markers were attached by the investigators. The marker placement protocol was based on the Vicon Plug-in Gait model and was further adapted according to relevant literature [14, 17, 19] and the technical characteristics of the RLRK. Markers were placed on the head, trunk, and lower limbs, for a total of 44 reflective markers (Table 2; Fig. 2). After marker placement, participants first practiced the RLRK on the force platform to familiarize themselves with the required standing position. Subsequently, on the technician’s verbal command, they performed the RLRK toward a dummy fitted with the Daedo electronic protector system. Testing was conducted first with the DS as the kicking leg, followed by the NS after a 30-s interval. The number of attempts was not fixed a priori. For each side, participants repeated the task until three valid scoring trials had been obtained. A trial was considered valid only when all of the following criteria were met: (1) the kicking foot contacted the predefined trunk target area of the dummy fitted with the Daedo electronic protector system; (2) the Daedo system recorded a valid score, which was required as an objective confirmation of effective target contact; (3) the movement was judged on site by a coach with sport-specific coaching experience to conform to the technical characteristics of the RLRK, including execution with the rear leg, a clear knee-lift action, rotation of the hip and trunk toward the target, a roundhouse kicking trajectory, and no obvious compensatory movement or loss of balance; and (4) the motion-capture and force-plate data were complete, without marker loss, obvious trajectory errors, or unsuccessful force-platform contact during the required events. Trials were excluded if the kick missed the target area, did not generate a valid Daedo score, involved obvious technical errors, stepping adjustments, loss of balance, or incomplete biomechanical data. The first three trials that met all validity criteria for each side were retained for biomechanical analysis, rather than selecting the highest-quality trials from a larger pool [19].

**Table 2:** Marker names and corresponding placement locations

Marker	Placement location	Marker	Placement location
RHAD	Right forehead	LHAD	Left forehead
RFAD	Right occipital region	LFAD	Left occipital region
C7	Spinous process of the 7th cervical vertebra	LASIS	Left anterior superior iliac spine
STRN	Manubrium of the sternum	LPSIS	Left posterior superior iliac spine
CLAV	Xiphoid process	LTROC	Left greater trochanter
T10	Spinous process of the 10th thoracic vertebra	LTH1-4	Four tracking markers on the left thigh
RASIS	Right anterior superior iliac spine	LLEP	Left lateral femoral epicondyle
RPSIS	Right posterior superior iliac spine	LMEP	Left medial femoral epicondyle
RTROC	Right greater trochanter	LSK1-4	Four tracking markers on the left shank
RTH1-4	Four tracking markers on the right thigh	LMLE	Left lateral malleolus
RLEP	Right lateral femoral epicondyle	LMME	Left medial malleolus
RMEP	Right medial femoral epicondyle	LHEEL	Left heel
RSK1-4	Four tracking markers on the right shank	LHM1	Left first metatarsophalangeal joint
RMLE	Right lateral malleolus	LHM5	Left fifth metatarsophalangeal joint
RMME	Right medial malleolus	RHM5	Right fifth metatarsophalangeal joint
RHEEL	Right heel	RHM1	Right first metatarsophalangeal joint

#### Phase definition

**Fig. 3:**
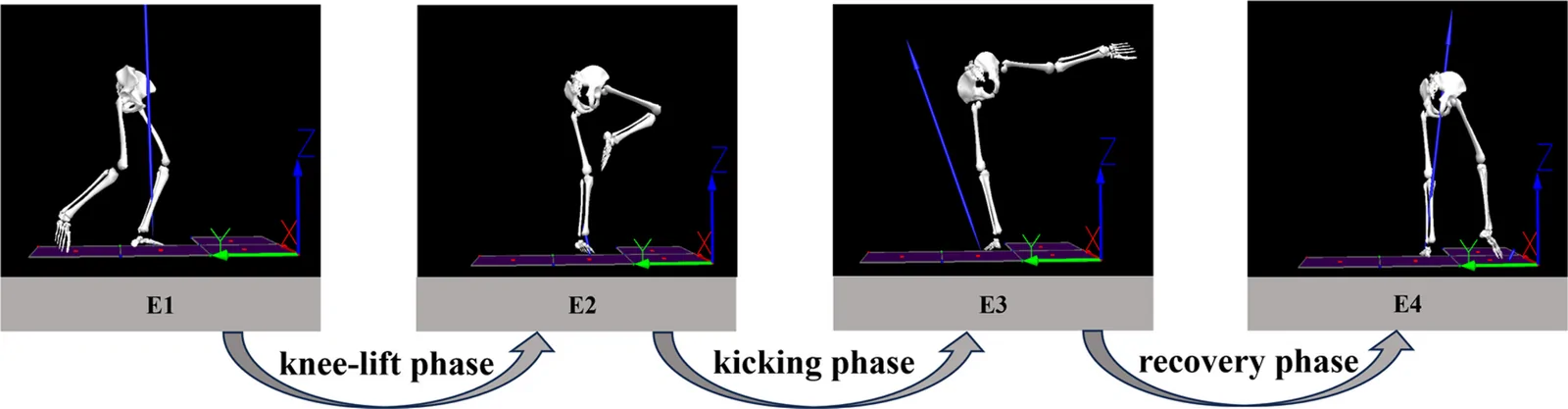
Schematic diagram of rear-leg roundhouse kick phase division

Based on the technical characteristics of the RLRK, relevant literature [2, 17, 20], and interviews with coaches and athletes, the movement was divided into four key events and three phases (Fig. 3; Supplementary Material 2). The kicking leg was defined as the lower limb used to strike the target, whereas the supporting leg was defined as the lower limb in contact with the ground and responsible for body-weight support.

E1 (preparation event) was defined as the instant at which both the supporting leg and kicking leg were in contact with the force platform and the kicking leg just began to leave the ground. E2 (knee-lift event) was defined as the instant of maximum knee flexion of the kicking leg. E3 (kicking event) was defined as the instant of maximum knee extension of the kicking leg during impact. E4 (recovery event) was defined as the instant at which the kicking leg contacted the force platform again during recovery, as indicated by the ground reaction force signal. Accordingly, the period from E1 to E2 was defined as the knee-lift phase, that from E2 to E3 as the kicking phase, and that from E3 to E4 as the recovery phase.

#### Data processing

**Fig. 4:**
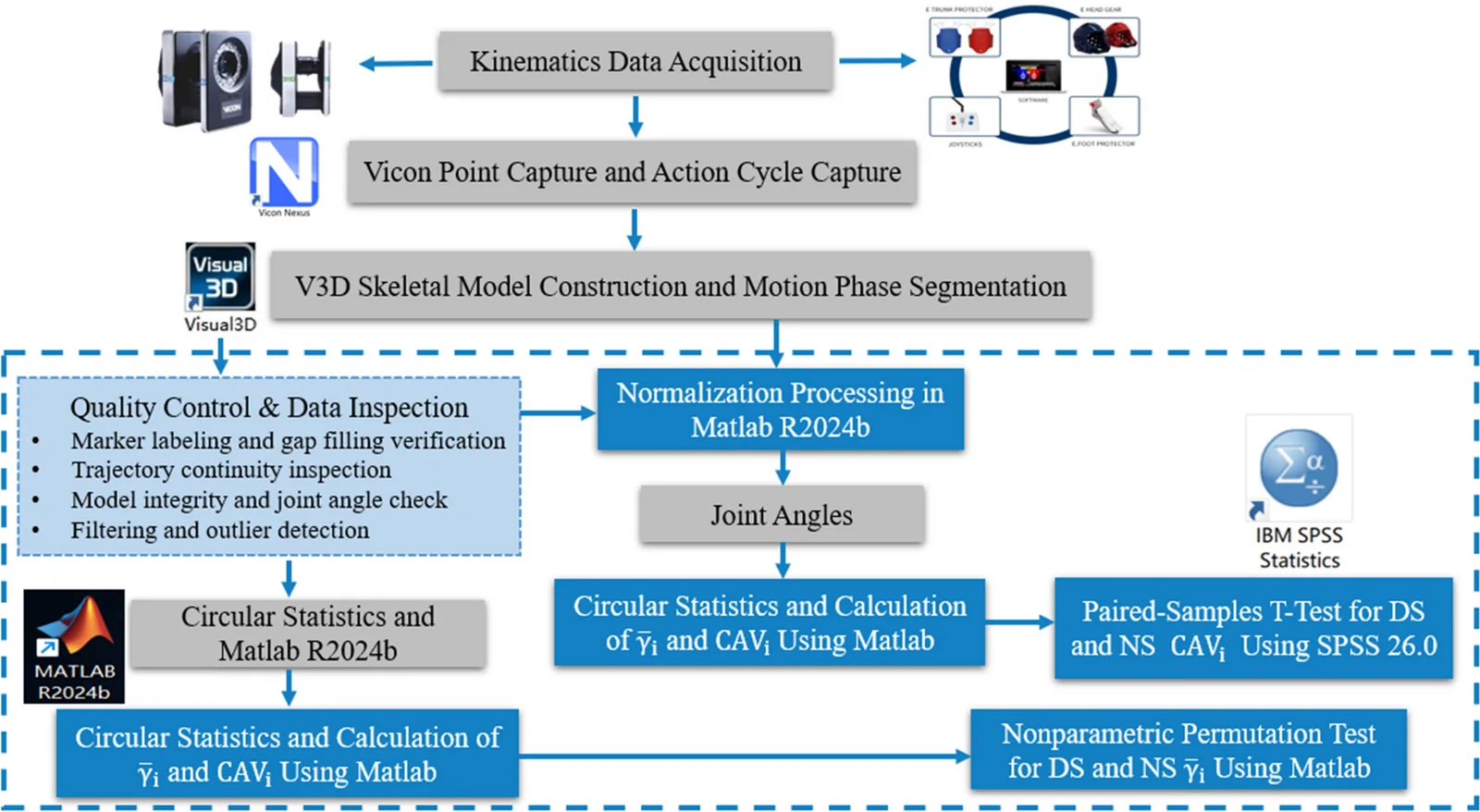
Data Processing Flowchart

As illustrated in Fig. 4, raw kinematic data were first preprocessed in Vicon Nexus 2.16 (Vicon, UK) and Visual3D 6.0 (C-Motion, USA). In Nexus, marker labeling and gap filling were completed, and all trials were visually inspected to confirm the continuity and completeness of marker trajectories. Trials containing tracking errors or excessive noise were excluded. A complete movement cycle was defined from E1 to E4. In Visual3D, a joint kinematic model was established, and both model integrity and the anatomical plausibility of joint angle outputs were checked. Marker trajectories were smoothed using a fourth-order Butterworth low-pass filter with a cutoff frequency of 15 Hz [2]. Each movement cycle was then time-normalized to 0–100%. Angle-angle plots were constructed for different joint couplings and planes of motion, with the proximal joint angle plotted on the horizontal axis and the distal joint angle on the vertical axis. A custom-written MATLAB script (R2024b; The MathWorks, Natick, MA, USA) was used to export and normalize lower-limb kinematic data. Before circular statistical analysis, additional data-quality control procedures were performed in MATLAB, including checks of joint-angle continuity and the identification of outliers. Inter-joint coordination was quantified using the vector coding technique. For each time point (*i*) in the normalized movement cycle, the CA (γᵢ) was calculated from consecutive proximal segment angles (${\uptheta}_{\mathrm{P}\left(\mathrm{i}\right)}$, ${\uptheta}_{\mathrm{P}\left(\mathrm{i}+1\right)}$) and consecutive distal segment angles (${\uptheta}_{\mathrm{D}\left(\mathrm{i}\right)}$, ${\uptheta}_{\mathrm{D}\left(\mathrm{i}+1\right)}$). according to Eqs. (1) and (2) [9]. The specific equations were as follows:

1 $$\begin{array}{l}{\upgamma}_{\mathrm{i}}=\mathrm{atan}\left(\frac{{\uptheta}_{\mathrm{D}\left(\mathrm{i}+1\right)}-{\uptheta}_{\mathrm{D}\left(\mathrm{i}\right)}}{{\uptheta}_{\mathrm{P}\left(\mathrm{i}+1\right)}-{\uptheta}_{\mathrm{P}\left(\mathrm{i}\right)}}\right)\cdot\frac{180}{\uppi},\\ {\uptheta}_{\mathrm{P}\left(\mathrm{i}+1\right)}-{\uptheta}_{\mathrm{P}\left(\mathrm{i}\right)}>0\end{array}$$

2 $$\begin{array}{l}{\upgamma}_{\mathrm{i}}=\mathrm{atan}\left(\frac{{\uptheta}_{\mathrm{D}\left(\mathrm{i}+1\right)}-{\uptheta}_{\mathrm{D}\left(\mathrm{i}\right)}}{{\uptheta}_{\mathrm{P}\left(\mathrm{i}+1\right)}-{\uptheta}_{\mathrm{P}\left(\mathrm{i}\right)}}\right)\cdot\frac{180}{\uppi}+180,\\ {\uptheta}_{\mathrm{P}\left(\mathrm{i}+1\right)}-{\uptheta}_{\mathrm{P}\left(\mathrm{i}\right)}<0\end{array}$$

subject to the following conditions [7]:

3 $${\upgamma}_{\mathrm{i}}=\left\{\begin{array}{ll}90,&\begin{array}{l}{\uptheta}_{\mathrm{P}\left(\mathrm{i}+1\right)}-{\uptheta}_{\mathrm{P}\left(\mathrm{i}\right)}=0\\ \text{and }{\uptheta}_{\mathrm{D}\left(\mathrm{i}+1\right)}-{\uptheta}_{\mathrm{D}\left(\mathrm{i}\right)}>0\end{array}\\ -90,&\begin{array}{l}{\uptheta}_{\mathrm{P}\left(\mathrm{i}+1\right)}-{\uptheta}_{\mathrm{P}\left(\mathrm{i}\right)}=0\\ \text{and }{\uptheta}_{\mathrm{D}\left(\mathrm{i}+1\right)}-{\uptheta}_{\mathrm{D}\left(\mathrm{i}\right)}<0\end{array}\\ -180,&\begin{array}{l}{\uptheta}_{\mathrm{P}\left(\mathrm{i}+1\right)}-{\uptheta}_{\mathrm{P}\left(\mathrm{i}\right)}<0\\ \text{and }{\uptheta}_{\mathrm{D}\left(\mathrm{i}+1\right)}-{\uptheta}_{\mathrm{D}\left(\mathrm{i}\right)}=0\end{array}\\ \mathrm{undefined},&\begin{array}{l}{\uptheta}_{\mathrm{P}\left(\mathrm{i}+1\right)}-{\uptheta}_{\mathrm{P}\left(\mathrm{i}\right)}=0\\ \text{and }{\uptheta}_{\mathrm{D}\left(\mathrm{i}+1\right)}-{\uptheta}_{\mathrm{D}\left(\mathrm{i}\right)}=0\end{array}\end{array}\right.$$

Using Eq. (4), the CA (γᵢ) was normalized to a range of 0°–360° [9].

4 $${\upgamma}_{\mathrm{i}}=\left\{\begin{array}{c}{\upgamma}_{\mathrm{i}}+360\hspace{0.25em}\hspace{0.25em}\hspace{0.25em}\hspace{0.25em},{\upgamma}_{\mathrm{i}}<0\\ {\upgamma}_{\mathrm{i}}\hspace{0.25em}\hspace{0.25em}\hspace{0.25em}\hspace{0.25em},{\upgamma}_{\mathrm{i}}\ge 0\hspace{0.25em}\hspace{0.25em}\hspace{0.25em}\hspace{0.25em}\end{array}\right.$$

Because CA is a directional vector variable, it was analyzed using circular statistics. The mean horizontal vector$\overline{\mathrm{x}}_{\mathrm{i}}$ and mean vertical vector$\overline{\mathrm{y}}_{\mathrm{i}}$were calculated according to Eqs. (5) and (6), respectively, and the mean CA $\left({\overline{\gamma }}_{i}\right)$ was subsequently obtained using Eq. (7) [9]. The mean CA was expressed as a circular mean, and its mean direction was determined from the average cosine and sine components of the CA using a quadrant-corrected arctangent equation, which is mathematically equivalent to the Atan2 function.

5 $${\overline{\mathrm{x}}}_{\mathrm{i}}=\frac{1}{\mathrm{n}}\sum_{\mathrm{i}=1}^{\mathrm{n}}\cos {\upgamma}_{\mathrm{i}}$$

6 $${\overline{\mathrm{y}}}_{\mathrm{i}}=\frac{1}{\mathrm{n}}\sum_{\mathrm{i}=1}^{\mathrm{n}}\sin {\upgamma}_{\mathrm{i}}$$

7 $${\overline{\upgamma}}_{\mathrm{i}}=\left\{\begin{array}{ll}\mathrm{atan}\left(\frac{{\overline{\mathrm{y}}}_{\mathrm{i}}}{{\overline{\mathrm{x}}}_{\mathrm{i}}}\right)\cdot\frac{180}{\uppi},&{\overline{\mathrm{x}}}_{\mathrm{i}}>0,\ {\overline{\mathrm{y}}}_{\mathrm{i}}\ge 0\\ \mathrm{atan}\left(\frac{{\overline{\mathrm{y}}}_{\mathrm{i}}}{{\overline{\mathrm{x}}}_{\mathrm{i}}}\right)\cdot\frac{180}{\uppi}+180,&{\overline{\mathrm{x}}}_{\mathrm{i}}<0\\ \mathrm{atan}\left(\frac{{\overline{\mathrm{y}}}_{\mathrm{i}}}{{\overline{\mathrm{x}}}_{\mathrm{i}}}\right)\cdot\frac{180}{\uppi}+360,&{\overline{\mathrm{x}}}_{\mathrm{i}}>0,\ {\overline{\mathrm{y}}}_{\mathrm{i}}<0\\ 90,&{\overline{\mathrm{x}}}_{\mathrm{i}}=0,\ {\overline{\mathrm{y}}}_{\mathrm{i}}>0\\ 270,&{\overline{\mathrm{x}}}_{\mathrm{i}}=0,\ {\overline{\mathrm{y}}}_{\mathrm{i}}<0\\ \mathrm{undefined},&{\overline{\mathrm{x}}}_{\mathrm{i}}=0,\ {\overline{\mathrm{y}}}_{\mathrm{i}}=0\end{array}\right.$$

According to Eq. (8), the resultant length of the mean CA $\left({\overline{\gamma }}_{i}\right)$was calculated [9].

8 $${\mathrm{r}}_{\mathrm{i}}=\sqrt{{{\overline{\mathrm{x}}}_{\mathrm{i}}}^{2}+{{\overline{\mathrm{y}}}_{\mathrm{i}}}^{2}}$$

According to Eq. (9), coupling angle variability ${\mathrm{CAV}}_{\mathrm{i}}$ was calculated [9].

9 $${\mathrm{CAV}}_{\mathrm{i}}=\sqrt{2\left(1-{\mathrm{r}}_{\mathrm{i}}\right)}\bullet \frac{180}{\uppi }$$

CA values were treated as circular variables in the statistical analysis. For each participant, circular means were first calculated across the three valid trials at each normalized time point to obtain an individual-level CA trajectory. Group-level CA trajectories were then derived using circular statistical methods. Given the circular nature of CA data, linear confidence intervals were not presented in the time-series plots; instead, coordination variability was quantified separately using established circular statistical indices.

The joint coordinate systems and joint angle definitions followed the default settings in Visual3D (Table 3). The three-dimensional hip joint angle was defined as the angle between the thigh and the line extending from the trunk, the three-dimensional knee joint angle as the angle between the shank and the line extending from the thigh, and the three-dimensional ankle joint angle as the angle between the foot and the line extending from the shank. Because the sagittal and frontal planes more directly capture the primary movement patterns and coordination characteristics of the lower-limb joints during the RLRK, only the coordination patterns and coordination variability of the bilateral hip, knee, and ankle joints in these two planes were analyzed in the present study.

**Table 3:** Lower-limb joint angles

Joint	Sagittal Plane (X)		Frontal Plane (Y)		Transverse Plane (Z)	
Hip joint	Flexion (+)	Extension (−)	Adduction (+)	Abduction (−)	Internal rotation (+)	External rotation (−)
Knee joint	Extension (−)	Flexion (+)	Adduction (+)	Abduction (−)	Internal rotation (+)	External rotation (−)
Ankle joint	Dorsiflexion (> 90°)	Plantarflexion (< 90°)	Inversion (+)	Eversion (−)	Internal rotation (+)	External rotation (−)

#### Angle-angle plots and coordination pattern classification

**Fig. 5:**
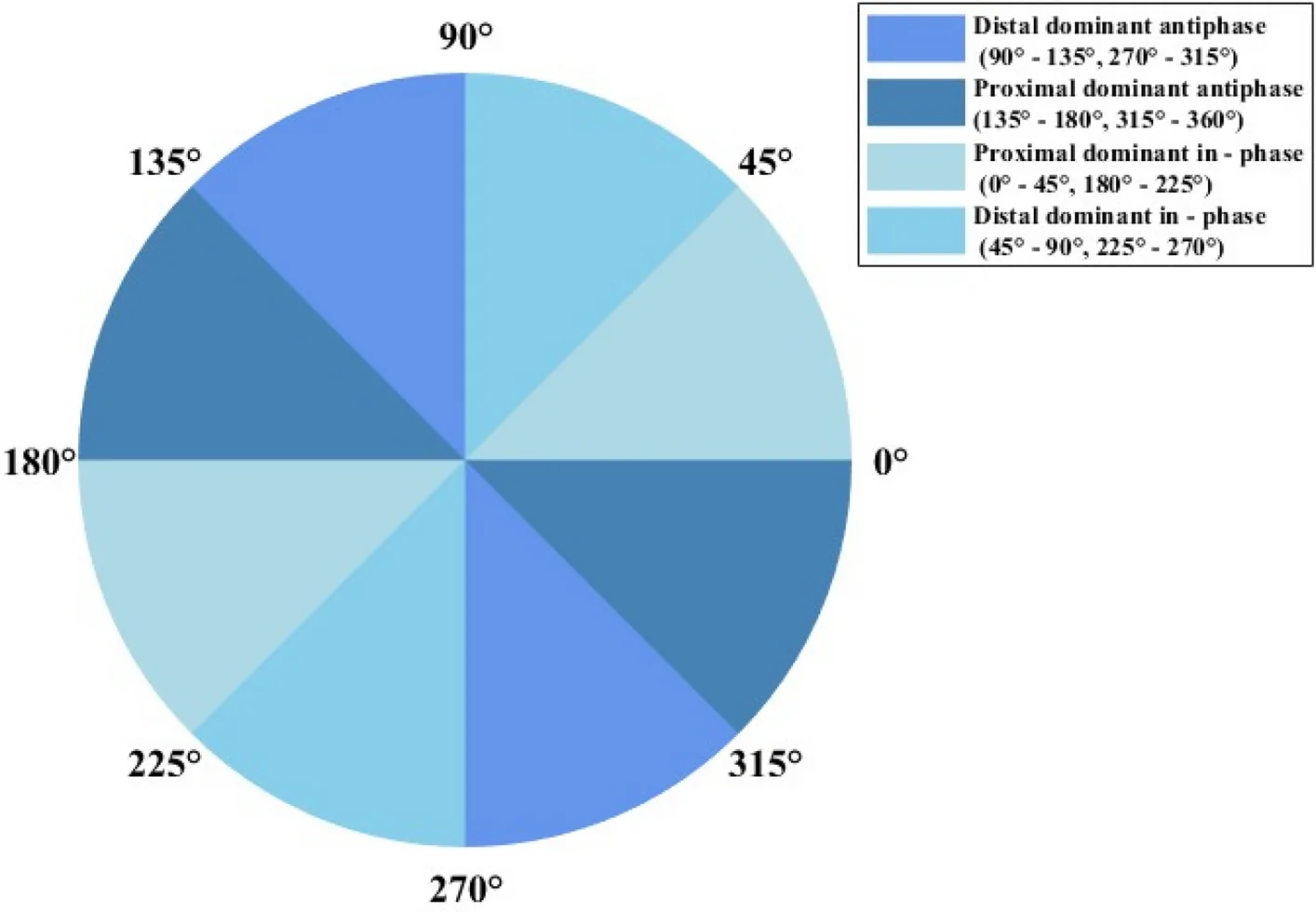
Phase Diagram of Joint Coupling Angle

The vector coding technique quantifies the continuously changing, time-varying interaction between segments by calculating the orientation of the vector connecting two consecutive points on an angle-angle plot relative to the positive *x*-axis. The resulting variable is the CA. After CA is mapped onto a polar coordinate space ranging from 0° to 360°, the movement can be classified into discrete joint coordination patterns. In the present study, the eight-sector framework proposed by Needham et al. [21] was used to define and identify joint coordination patterns during the RLRK. Under this framework, inter-joint coordination was interpreted as four coordination modes: in-phase with proximal dominance, in-phase with distal dominance, anti-phase with distal dominance, and anti-phase with proximal dominance. These modes were used to characterize bilateral differences in multi-joint coordinative control across different movement phases (Fig. 5).

As illustrated in Fig. 5, the CA is categorized into distinct coordination modes based on its magnitude (Table 4).

**Table 4:** Joint coordination patterns

Coordination mode	CA Range	Movement Pattern Description
*Proximal dominant in-ph*ase (PI)	0° < CA < 45°; 180° < CA < 225°	Proximal and distal coextensive motion, proximal motion is large in amplitude
Distal dominant in-phase (DI)	45° < CA < 90°; 225° < CA < 270°	Proximal and distal in the same direction, distal motion large amplitude
Distal dominant anti-phase (DA)	90° < CA < 135°; 270° < CA < 315°	Proximal and distal reverse motion, distal motion large amplitude
Proximal dominant anti-phase (PA)	135° < CA < 180°; 315° < CA < 360°	Proximal and distal reverse motion, proximal motion large amplitude
	0° or 180°	Distal end fixed, proximal end active
	90° or 270°	Proximal end fixed, distal end active
	45° or 225°	Proximal and distal ends move in the same direction and have the same amplitude of motion
	135° or 315°	Proximal and distal reverse motion with same amplitude of motion

### Statistical analysis

Data were processed in SPSS 26.0 and MATLAB R2024b (CircStat toolbox) [22]. Bilateral differences in CA were examined in MATLAB using a non-parametric permutation test [23]. Bilateral differences in coordination variability were analyzed in SPSS 26.0. Normality was assessed using the Shapiro–Wilk test. If the data were normally distributed, a paired-samples *t*-test was applied; otherwise, the Wilcoxon signed-rank test was used. Results were expressed as mean ± standard deviation (x̄ ± s). The level of statistical significance was set at *p* < 0.05, where * indicates *p* < 0.05 and ** indicates *p* < 0.01. Cohen’s *d* was used to evaluate effect size, with thresholds defined as small (0.2 ≤ *d* < 0.5), medium (0.5 ≤ *d* < 0.8), and large (*d* ≥ 0.8) [24].

## Results

### Characteristics of inter-joint coordination organization in the lower limbs

**Fig. 6:**
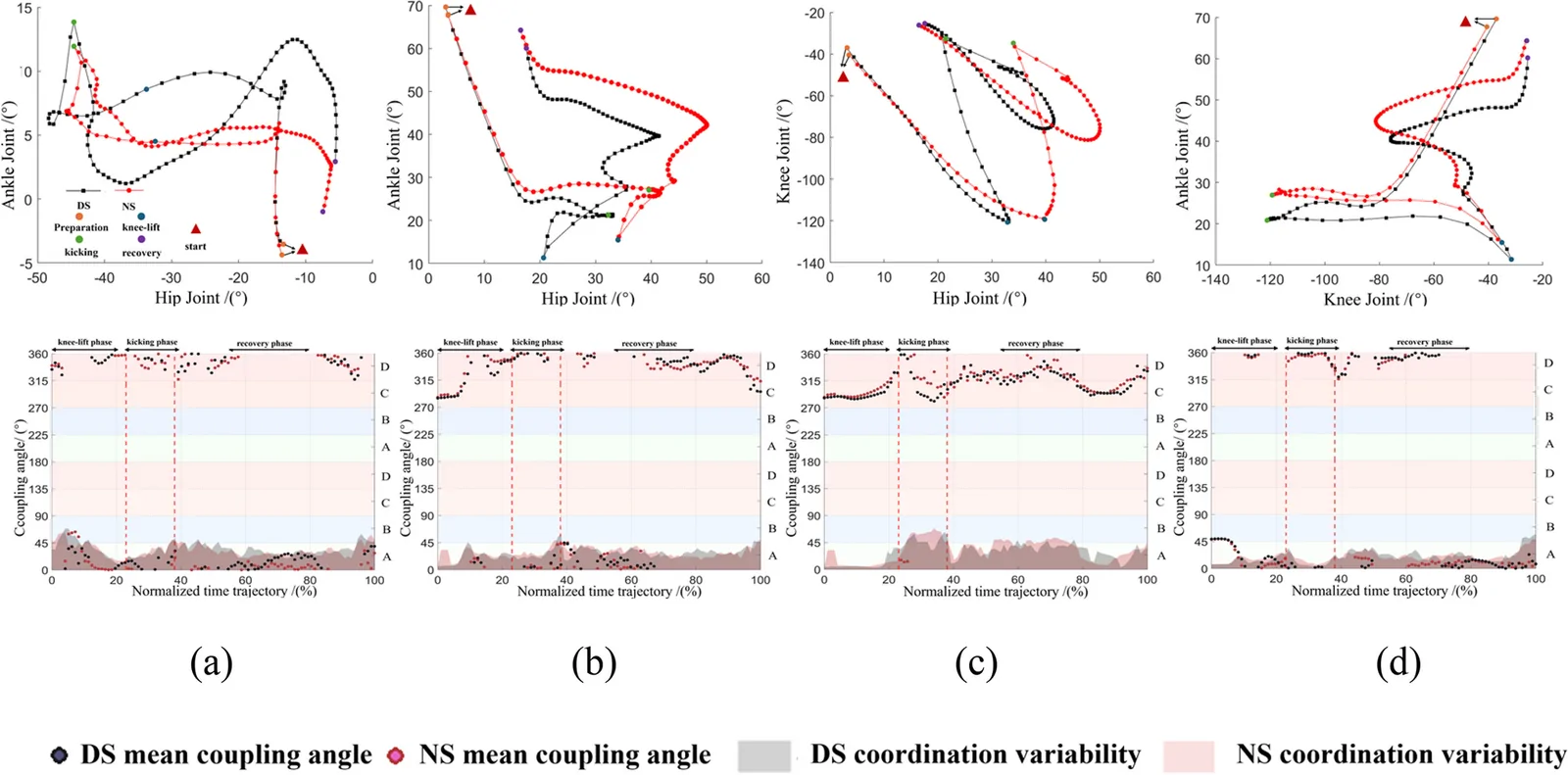
Angle-angle plots and coupling angle time-series plots. Note: In the Angle-angle plots and coupling angle time-series plots a, b, c, d denote Hip-ankle coupling in the frontal plane Hip-ankle coupling in the sagittal plane. Hip-knee coupling in the sagittal plane. Knee-ankle coupling in the sagittal plane. In the coupling angle time-series plots A, B, C, D denote in-phase with proximal dominance, in-phase with distal dominance, anti-phase with distal dominance, and anti-phase with proximal dominance, respectively

As shown in Fig. 6, differences in inter-joint coordination organization between the bilateral RLRK were observed across movement phases. During the knee-lift phase, the overall coordination organization of the two sides were relatively similar, with the main difference observed in the hip-ankle coupling in the frontal plane. No marked bilateral differences were observed in most joint couplings during this phase, whereas the frontal-plane hip–ankle coupling showed different coupling-angle characteristics between the DS and NS. During the kicking phase, bilateral differences in coordination organization were mainly observed, particularly in the hip-ankle coupling in the frontal plane and the knee-ankle coupling in the sagittal plane. The coupling-angle characteristics of these two joint couplings differed between the DS and NS, indicating that the bilateral differences shown in Fig. 6 were mainly concentrated in the kicking phase. During the recovery phase, the overall coordination organization of the two sides became similar again. However, bilateral differences were still observed in the knee-ankle coupling in the sagittal plane, as reflected by different coupling-angle characteristics between the DS and NS.

### Comparison of mean coupling angles between the DS and NS across movement phases

**Fig. 7:**
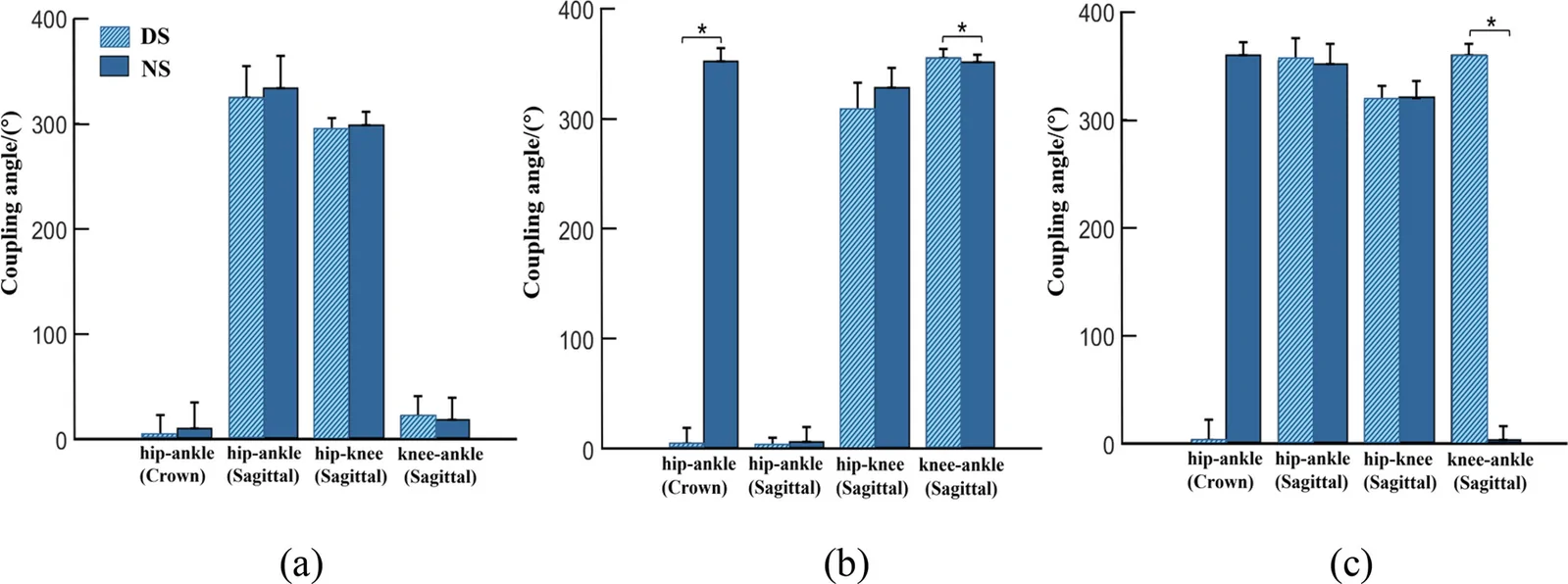
Mean coupling angles of the NS and DS in the sagittal and frontal planes across different movement phases. **a** Knee-lifting phase, **b** kicking phase, **c** recovery phase note: * *p* < 0.05, ** *p* < 0.01

As shown in Fig. 7, during the knee-lift phase, no significant bilateral differences were found in the mean CA for any joint coupling. During the kicking phase, significant bilateral differences were observed in the mean CA of the hip-ankle coupling in the frontal plane (DS: 4.92° ± 14.79°; NS: 352.83° ± 12.04°; *p* = 0.037, *d* = 0.89) and the knee-ankle coupling in the sagittal plane (DS: 355.06° ± 8.26°; NS: 351.11° ± 7.73°; *p* = 0.026, *d* = 0.73).During the recovery phase, a significant bilateral difference was found in the mean CA of the knee-ankle coupling in the sagittal plane (DS: 359.33° ± 11.93°; NS: 2.82° ± 12.88°; *p* = 0.016, *d* = 0.72).

### Comparison of lower-limb joint coordination variability between the DS and NS

**Fig. 8:**
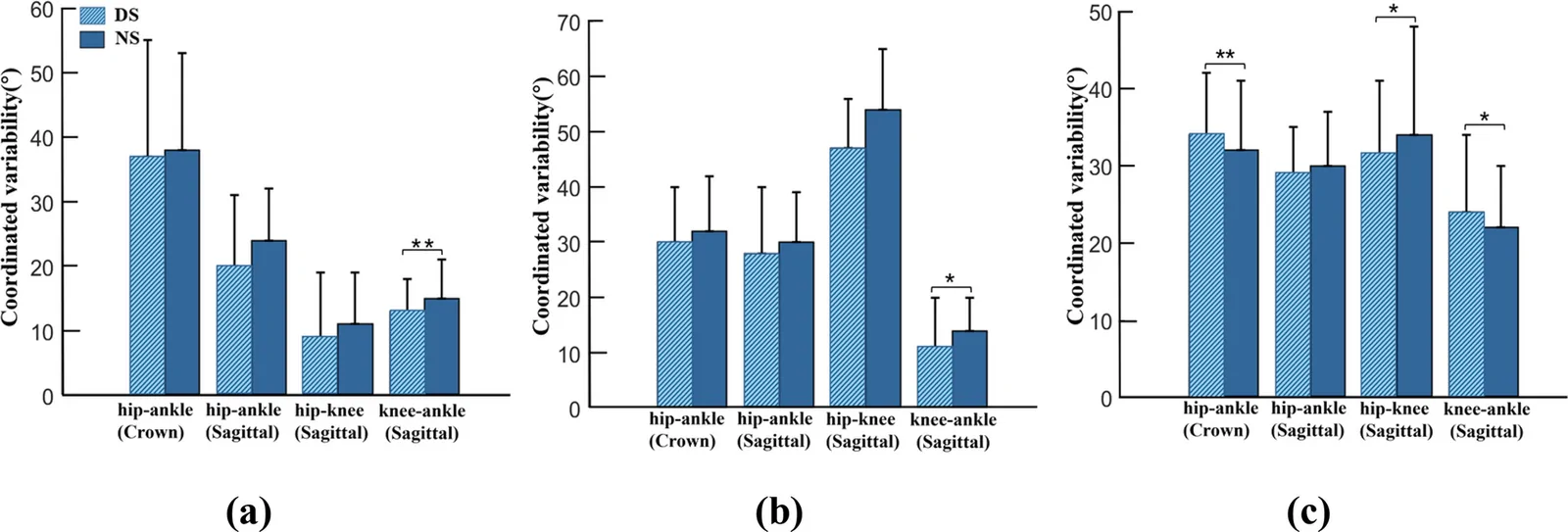
Coordination variability of the NS and DS in the sagittal and frontal planes across different movement phases. **a** Knee-lifting phase, **b** kicking phase, **c** recovery phase. Note: * *p* < 0.05, ** *p* < 0.01

As shown in Fig. 8, during the knee-lift phase, a significant bilateral difference was observed in the coordination variability of the knee-ankle coupling in the sagittal plane, with the DS showing lower variability than the NS (DS: 13° ± 5°; NS: 15° ± 6°; *p* = 0.002, *d* = 0.78).During the kicking phase, a significant bilateral difference was also found in the coordination variability of the knee-ankle coupling in the sagittal plane, with the DS showing lower variability than the NS (DS: 11° ± 9°; NS: 14° ± 6°; *p* = 0.015, *d* = 0.75).During the recovery phase, significant bilateral differences were observed in the coordination variability of the hip-ankle coupling in the frontal plane, the hip-knee coupling in the sagittal plane, and the knee-ankle coupling in the sagittal plane. Specifically, coordination variability of the hip-ankle coupling in the frontal plane was higher on the DS than on the NS (DS: 35° ± 8°; NS: 32° ± 9°; *p* = 0.008, *d* = 0.59), coordination variability of the hip-knee coupling in the sagittal plane was lower on the DS than on the NS (DS: 31° ± 10°; NS: 34° ± 14°; *p* = 0.027, *d* = 0.66), and coordination variability of the knee-ankle coupling in the sagittal plane was higher on the DS than on the NS (DS: 24° ± 10°; NS: 22° ± 8°; *p* = 0.044, *d* = 0.59).

## Discussion

### Characteristics of lower-limb joint coordination organization

The present study found clear side-related differences in lower-limb joint coordination organization between the DS and NS during the RLRK in competitive taekwondo. These differences were not primarily reflected in whether the two sides were able to complete a similar technical action, but rather in the coupling-angle characteristics and coordination organization observed across different movement phases. Overall, coordination organization were relatively similar between the two sides during the knee-lift phase, suggesting a certain degree of commonality in the preparatory organization of the movement. Bilateral differences were most pronounced during the kicking phase, indicating that side-related differences were mainly concentrated in the key output stage of the action. During the recovery phase, different local coupling characteristics were still retained despite a similar task goal, suggesting that the final stage of the movement also involves specific temporal organization of joint motion rather than representing a purely passive return process.

During the kicking phase, clear bilateral differences were observed in the hip-ankle coupling in the frontal plane and the knee-ankle coupling in the sagittal plane, indicating that the DS-NS difference was not restricted to an isolated joint but was more likely expressed in the local organization of inter-joint coupling during the key output phase of the movement. Specifically, the DS showed different coupling-angle characteristics, characterized by continuous ankle eversion-inversion adjustments during hip abduction–adduction in the frontal plane and by multi-stage ankle dorsiflexion-plantarflexion alternations accompanying knee extension in the sagittal plane. By contrast, the NS displayed less pronounced changes in these coupling-angle characteristics, namely an eversion-inversion mode in the frontal plane and persistent plantarflexion during knee extension in the sagittal plane. These findings suggest that, under the same overall task demand, the DS and NS exhibited side-specific kinematic coordination organization in both planes, rather than indicating that one side necessarily demonstrated superior or inferior coordination control.

This finding is generally consistent with previous research on lateral asymmetry in kicking performance. Earlier studies have shown that, in techniques such as the side kick and double roundhouse kick, significant differences may exist between the DS and NS in variables including kicking-leg kinetics, angular velocity, linear velocity, linear momentum, and ground reaction force [14, 18]. Studies on scoring performance in the front kick and double roundhouse kick have also indicated that local joint parameters of the kicking leg during the kicking phase are closely related to distal output performance [14, 20]. In addition, previous work has suggested that different kicking techniques may exhibit distinct hip-knee coordination patterns, and that kicking speed is closely associated with inter-joint coordination characteristics [25]. A comparative study of high-impact and low-impact roundhouse kick further reported that kicks with greater impact effectiveness were often characterized by smaller ankle plantarflexion angles before and at the instant of impact, together with different muscle activation features [26]. Collectively, these studies indicate from the perspectives of discrete kinematics, kinetics, and electromyography that bilateral differences in kicking actions objectively exist and may be related to local joint organization. However, it should be noted that most existing studies have focused on peak angles, velocities, kinetic variables, or electromyographic activity, whereas direct evidence regarding continuous coupling characteristics remains limited. Therefore, the specific coupling-angle characteristics observed in the present study cannot yet be directly matched item by item with previous findings. Research from other kicking sports has further suggested that kicking actions are characterized by a proximal-to-distal segmental organization, and that differences between the preferred and non-preferred sides may also exist in terminal foot velocity, knee joint moment, supporting-leg kinetics, as well as frontal-plane kinematics and muscle activation timing of the supporting limb [27–31]. Taken together, the available evidence supports the existence of bilateral asymmetry in kicking tasks, but remains insufficient to directly account for the specific phase-dependent coordination organization identified in the present study.

Based on the findings of the present study and the existing evidence, the multi-stage hip-ankle coupling adjustments and distal modulation of the knee-ankle coupling observed on the DS during the kicking phase may indicate side-specific kinematic coordination organization during the distal output stage of the movement. In contrast, the coupling-angle characteristics observed on the NS may reflect a different form of inter-joint coordination organization when performing the same kicking task. These interpretations are based on mean CA trajectories and coordination variability, rather than on quantified time proportions or transition frequencies of specific coordination modes. Based on the currently available evidence, these bilateral differences may be associated with task-specific lateral adaptations developed through long-term sport-specific training. However, because the present study was based on kinematic data only, these findings should be interpreted as differences in movement coordination organization rather than as direct evidence of neuromuscular control mechanisms. Further studies combining kinetic, electromyographic, and intervention-based approaches are needed to verify the underlying mechanisms.

Compared with the kicking phase, the recovery phase was characterized by greater similarity between the DS and NS in certain movement patterns, although clear differences remained in the overall coordination organization. The present study showed that the movement pattern of the knee-ankle coupling in the sagittal plane was broadly similar on both sides. Specifically, both the DS and NS began with knee flexion accompanied by ankle plantarflexion, followed by a sequence of knee flexion–extension-flexion–extension with sustained ankle dorsiflexion, suggesting a relatively high degree of task commonality during this phase. However, at the level of overall coordination organization, the DS and NS still showed different coupling-angle characteristics. In addition, the knee flexion angle was smaller on the DS than on the NS. Together, these findings suggest that the recovery phase should not be regarded as a purely mechanical repositioning process after impact, but rather as a movement stage involving specific temporal organization of joint motion.

Existing evidence indicates that joint coordination and coordination variability can change with preparation time and task demands in unanticipated change-of-direction tasks [32]. Under different walking surface conditions, the synchrony and variability of ankle-knee and knee-hip coordination patterns have also been shown to adjust, and uneven or sloped surfaces may even induce more in-phase or more variable coordination patterns [33, 34]. In taekwondo-related research, the recovery phase of the roundhouse kick has likewise been regarded as more than a purely passive return movement, as it is still accompanied by substantial muscle activation and coordination requirements. Different kicking heights may also alter the movement pattern and electromyographic response of the supporting leg during the recovery phase [35, 36]. In addition, target distance and competitive level have been reported to influence taekwondo kicking performance [37, 38]. Collectively, these studies suggest that coordination organization and coordination variability in the terminal stage of movement are influenced by task constraints, environmental conditions, and movement requirements. However, the specific coupling-angle characteristics observed in the present study cannot yet be directly matched to the existing literature. The main reason is that prior studies have focused primarily on movement outcomes, reaction time, and discrete kinematic, kinetic, or electromyographic variables, whereas direct evidence on continuous coupling characteristics during the recovery phase remains limited. Therefore, the current literature supports the view that the recovery phase warrants discussion from the perspective of movement coordination organization, but remains insufficient to provide a direct explanation for the specific joint coupling characteristics observed in the present study.

Based on these findings, it appears that the DS and NS do not rely on identical temporal organizations of joint motion when performing a similar recovery task. Despite the broad similarity in movement pattern, the DS and NS showed different coupling-angle characteristics, indicating that the two sides may exhibit different temporal organizations of inter-joint coordination during the leg-withdrawal and re-contact process. Relatively speaking, the DS may involve different joint-coupling organization from the NS during this phase, but this should not be interpreted as evidence that one side adopted a superior recovery strategy.

Overall, the differences between the DS and NS during the RLRK showed clear phase-specific characteristics. In the kicking phase, bilateral differences were mainly reflected in the local organization of inter-joint coupling associated with distal output, whereas in the recovery phase they were primarily reflected in differences in the temporal organization of joint motion under broadly similar movement patterns. Whereas previous studies have mainly revealed lateral asymmetry in kicking actions through discrete kinematic, kinetic, and electromyographic variables, the present findings suggest that lower-limb side-related differences may be expressed less as isolated differences in peak joint parameters and more as differences in multi-joint coordination organization, phase-related coupling-angle characteristics, and inter-joint coordination variability. Accordingly, the distinction between the DS and NS should not be reduced to a simple difference in single-joint function, but rather interpreted as a side-specific feature of multi-joint coordinative organization. This asymmetry may be associated with task-specific adaptations developed through long-term sport-specific training, although the underlying mechanisms still require further verification through kinetic, electromyographic, and intervention-based studies.

### Characteristics of lower-limb joint coordination variability

Coordination variability is generally understood as the range and pattern of fluctuation in the coordination relationship between joints or segments when an individual repeatedly performs the same goal-directed movement [39]. This variable is commonly quantified using methods such as vector coding and continuous relative phase, and is used to characterize the temporal changes and variability level of joint or segment coupling during movement execution. It is therefore a widely used indicator for evaluating the organizational characteristics of motor coordination [9, 40, 41]. Traditionally, variability has often been regarded as movement error or control noise. However, recent research in motor control has increasingly suggested that an appropriate degree of coordination variability reflects the adaptability and flexibility of the movement system, thereby helping individuals cope with environmental perturbations, changes in task constraints, and momentary movement errors [6, 42, 43]. Therefore, the biological significance of coordination variability is highly task-dependent: an increase in variability does not necessarily indicate poorer movement organization, nor does a decrease necessarily indicate better movement organization, but instead should be interpreted in relation to the movement phase, task goal, and functional role of the specific joint coupling involved [44]. Based on this perspective, the present discussion of coordination variability between the DS and NS is primarily grounded in the phase-specific demands of the RLRK, rather than making deterministic inferences about its underlying mechanisms.

The present study found that differences in coordination variability between the DS and NS during the RLRK were clearly phase-dependent. During the knee-lift and kicking phases, coordination variability was lower in certain joint couplings on the DS than on the NS, with a significant bilateral difference observed in the knee-ankle coupling in the sagittal plane. In light of the task characteristics of these phases, the knee-lift and kicking phases represent the key stages for force preparation, momentum transfer, and distal output during the RLRK, and therefore place relatively high demands on movement timing, output consistency, and local coordination precision. Lower coordination variability on the DS may indicate a more repeatable coupling organization under the present task constraints, whereas the higher variability observed on the NS may indicate a greater range of coupling fluctuation when performing the same task. However, this should not be interpreted as direct evidence that the DS had superior motor control or that the NS had poorer motor control. Rather, it indicates that the two sides showed different levels of coupling fluctuation under the specific task demands of these phases. Previous studies have shown that the interpretation of movement variability and coordination variability depends on the specific task, and that task complexity, constraint conditions, and changes in movement organization can all influence coordination variability [32, 45, 46]. At the same time, some studies of unilateral functional tasks in healthy individuals have not found a significant effect of limb dominance on lower-limb coordination variability [40, 47]. This suggests that the DS-NS differences observed in the present study are more appropriately understood as phase-specific differences in kinematic coordination organization during the key output stage, rather than as direct evidence of different neuromuscular control strategies.

Unlike the knee-lift and kicking phases, differences in coordination variability during the recovery phase did not show a uniform directional pattern, but instead exhibited clear joint-specific characteristics. During this phase, coordination variability was higher on the DS than on the NS in some multi-joint couplings, with significant bilateral differences observed in the hip-ankle coupling in the frontal plane and the knee-ankle coupling in the sagittal plane. In contrast, in other joint couplings, such as the hip-knee coupling in the sagittal plane, coordination variability on the DS was lower than that on the NS. These findings suggest that bilateral differences during the recovery phase cannot be simply summarized as “higher on the DS” or “lower on the DS,” but should instead be interpreted as reflecting different functional roles of joint couplings in the terminal stage of the movement. Previous studies on lower-limb coordination have shown that environmental and task constraints can substantially influence coordination patterns and their variability. In walking studies, Ippersiel et al. (2021,2022) [33, 34] reported that uneven surfaces can induce greater coordination variability and more synchronized coordination patterns, whereas some more conservative movement organizations may be associated with lower coordination variability. At the same time, the optimal variability perspective proposed by Stergiou et al.(2006) [42] suggests that higher variability does not necessarily indicate poorer movement organizations, but may instead reflect greater flexibility for adjustment in the final stage of movement; conversely, lower variability does not necessarily indicate superior movement organizations, but may correspond to a more fixed and conservative coordination strategy. In light of the present findings, the DS exhibited higher coordination variability in some distal-related joint couplings, while showing lower variability than the NS in some proximal-related joint couplings. This suggests that the side-related difference during the recovery phase of the RLRK is better understood as a structural distribution difference in coordination variability across joint couplings, rather than as a simple difference in overall magnitude.

Overall, the phase-specific differences in coordination variability between the DS and NS during the RLRK suggest that the two sides do not rely on an identical kinematic coordination organization throughout the movement. During the knee-lift and kicking phases, lower coordination variability in certain joint couplings on the DS is more likely to reflect a more repeatable coupling organization during the key output stages of the action, whereas the higher coordination variability observed on the NS suggests a greater range of coupling fluctuation. This interpretation does not imply that lower variability necessarily represents greater stability or better control, or that higher variability necessarily reflects poorer control. Upon entering the recovery phase, the bilateral difference further shifted toward joint-specific directional divergence, indicating that the significance of coordination variability cannot be adequately summarized by a simple higher-versus-lower relationship. In the context of the present study, the difference between the DS and NS should not be interpreted as simply “better” or “worse,” but rather as a difference in coordination organization across movement phases and joint couplings. Because the present analysis was based solely on kinematic variables, it is not yet possible to draw further conclusions regarding the underlying neuromuscular control mechanisms, kinetic cost, or trainability of these differences. Accordingly, these interpretations still require verification through kinetic, surface electromyographic, and intervention-based studies.

From a training-practice perspective, the present findings may provide kinematic references for non-dominant-side technical training and bilateral coordination training. In particular, the observed DS–NS differences in hip-ankle and knee-ankle coupling suggest that coaches may pay attention to side-related differences in inter-joint coordination during the knee-lift, kicking, and recovery phases. However, the goal of training is not simply to decrease or increase coordination variability per se, but rather as improving the task-specific organization of movement variability across different phases and joint couplings. Because the present study did not include intervention training, performance outcomes, injury records, kinetic loading, or electromyographic evidence, these findings should not be interpreted as direct evidence that a specific training strategy can improve performance or reduce injury risk. Instead, they provide a kinematic basis for future studies examining the relationships among bilateral coordination characteristics, sport-specific performance, and injury-related factors.

### Limitations

This study has several limitations. First, only male taekwondo athletes were included, and therefore the generalizability of the findings to female athletes remains to be established. Second, the present analysis focused primarily on coordination patterns and coordination variability between the DS and NS based on kinematic variables, without the concurrent inclusion of kinetic or sEMG measures. Consequently, the present findings should be interpreted primarily as kinematic evidence of coordination organization, and further interpretation of the underlying neuromuscular control mechanisms and mechanical implications remains limited. Third, bilateral differences were mainly described in terms of CA trajectories and coordination variability, whereas the time proportion and transition frequency of different coordination patterns were not further quantified. Therefore, the interpretation of inter-joint coordination organization in this study should be understood mainly based on mean CA and coordination variability, rather than the detailed temporal distribution of specific coordination modes. Future studies may further quantify the relative time spent in different coordination modes and their transition characteristics across movement phases. Future research should incorporate larger samples, include female athletes, and combine kinetic, sEMG, and intervention-based approaches to more systematically examine the mechanisms underlying bilateral coordination differences during the RLRK and their implications for sport-specific training.

## Conclusions

When competitive taekwondo athletes performed the RLRK with the DS and NS, phase-specific differences were observed in lower-limb inter-joint coordination patterns and coordination variability. These differences were most evident during the kicking and recovery phases and were mainly reflected in the organization of hip-ankle, hip-knee, and knee-ankle joint couplings. Overall, the DS–NS differences should not be interpreted simply as “better” or “worse,” but rather as side-specific differences in multi-joint coordination organization across movement phases. The present findings provide kinematic evidence for understanding bilateral coordination characteristics during the RLRK and may serve as a reference for future studies and sport-specific technical assessment.

## Supplementary Information

Supplementary Material 1.

Supplementary Material 2.

## Data Availability

The datasets generated and/or analysed during the current study are available from the corresponding author on reasonable request.

## Abbreviations

CA: Coupling angle
CAV: Coupling angle variability
DS: Dominant side
NS: Non-dominant side
RLRK: Rear-leg roundhouse kick
sEMG: Surface electromyography
