# Supplementary material for: Lower-limb coordination during the rear-leg roundhouse kick in competitive taekwondo: a vector-coding analysis
Source: BMC Sports Sci Med Rehabil. 2026 Jul 31;18:528. doi: 10.1186/s13102-026-01831-5 (PMC13715184; doi:10.1186/s13102-026-01831-5)
Supplement: Supplementary file 1 [file 13102_2026_1831_MOESM1_ESM.pdf]

Dear Participants:

Thank you for participating in our study on the rear-leg roundhouse kick in taekwondo. Before taking part in the experiment, we would like to ensure that you fully understand the purpose, procedures, and potential risks involved. Please read the following information carefully. If you agree to participate, please sign this informed consent form.

Experiment Description:

The purpose of this study is to investigate the characteristics of bilateral lower-limb motor coordination during the execution of the rear-leg roundhouse kick in competitive taekwondo athletes. During the experiment, data will be collected from three effective scoring rear-leg roundhouse kicks performed with the dominant and nondominant lower limbs, respectively. The experiment may involve the following procedures:

1. **Marker placement:** Forty-four reflective markers will be attached to both lower limbs.
2. **Movement execution:** Participants will perform rear-leg roundhouse kicks against an electronic protector on a standard competition mat. The dominant and nondominant sides will each be tested, and three effective scoring kicks will be collected for each side. A qualified coach will be present on site to evaluate whether the tested movements comply with the technical standards of the rear-leg roundhouse kick.
3. **Data analysis:** The collected data will be used for research and analysis in the field of sports training to explore the characteristics of bilateral lower-limb movement coordination during the execution of the rear-leg roundhouse kick in competitive taekwondo athletes.

Risks and Benefits:

Participation in this experiment may involve certain risks, including but not limited to minor discomfort caused by the measuring equipment on the skin, fatigue, or discomfort during physical activity. However, we will take all necessary measures to ensure your safety and minimize any potential risks. At the same time, your participation will contribute to scientific research and may help advance sports training practice.

Confidentiality and Privacy:

We will strictly comply with relevant laws and regulations concerning the protection of personal information and will take appropriate measures to safeguard your privacy. The data collected during the experiment will be used solely for research purposes and may be anonymously published or shared within the academic community.

Voluntary Participation:

Your participation in this experiment is entirely voluntary. You have the right to withdraw from the experiment at any time without any negative consequences. You may also contact us to withdraw your consent and request the deletion of your personal information.

If you agree to participate in this experiment and have understood the information provided above, please sign your name and date below. If you have any questions or need further clarification, please feel free to contact us.

Thank you for your cooperation and support.

Participant’s Name: ______________________ Date: ______________________

徐嘉良	崔润泽	宗毅	章屿.
方润统	高子浩	杨元行	孙靖博.
杨斌亿	张庆泰	蒋志豪	聂雨田
