# Supplementary material for: Lower-limb coordination during the rear-leg roundhouse kick in competitive taekwondo: a vector-coding analysis
Source: BMC Sports Sci Med Rehabil. 2026 Jul 31;18:528. doi: 10.1186/s13102-026-01831-5 (PMC13715184; doi:10.1186/s13102-026-01831-5)
Supplement: Supplementary file 2 [file 13102_2026_1831_MOESM2_ESM.pdf]

Expert Interview on Phase Division of the Taekwondo Rear-Leg Roundhouse Kick

Interview Topic

Phase division of the taekwondo rear-leg roundhouse kick (RLRK)

Interviewees

Jialiang Xu, Runze Cui, Yi Zong, Yu Zhang, et al.

Researcher

Yong Ma

Interview Date and Location

November 30, 2020, Exercise Efficacy Evaluation Laboratory, Wuhan Sports University

Interview Record

To determine the phase division of the taekwondo rear-leg roundhouse kick (RLRK), expert interviews were conducted with experienced coaches and athletes. Based on the technical characteristics of the RLRK, the movement was divided into four key events and three phases. In this movement, the kicking leg was defined as the lower limb used to strike the target, whereas the supporting leg was defined as the lower limb in contact with the ground and responsible for body-weight support.

The four key events were defined as follows:

E1 (preparation event): the instant at which both the supporting leg and kicking leg were in contact with the force platform and the kicking leg had just begun to leave the ground.

E2 (knee-lift event): the instant of maximum knee flexion of the kicking leg.

E3 (kicking event): the instant of maximum knee extension of the kicking leg during impact.

E4 (recovery event): the instant at which the kicking leg contacted the force platform again during recovery, as indicated by the ground reaction force signal.

Accordingly, the movement was divided into three phases:

Knee-lift phase (E1–E2): from the preparation event to the knee-lift event. This phase mainly involved lifting the kicking leg and preparing for the subsequent strike.

Kicking phase (E2–E3): from the knee-lift event to the kicking event. This phase was characterized by rapid extension of the kicking leg to complete the strike and maximize impact effectiveness.

Recovery phase (E3–E4): from the kicking event to the recovery event. This phase mainly involved retracting the kicking leg and re-establishing body stability after impact.

The phase division obtained from the expert interviews was used as the basis for subsequent motion-event identification and phase-based biomechanical analysis in the present study.
